# Two Self-Incompatibility Sites Occur Simultaneously in the Same *Acianthera* Species (Orchidaceae, Pleurothallidinae)

**DOI:** 10.3390/plants9121758

**Published:** 2020-12-11

**Authors:** Mariana Oliveira Duarte, Denise Maria Trombert Oliveira, Eduardo Leite Borba

**Affiliations:** Departamento de Botânica, Instituto de Ciências Biológicas, Universidade Federal de Minas Gerais, Av. Pres. Antônio Carlos, 6627, Pampulha, Belo Horizonte 31270-901, Minas Gerais, Brazil; dmtoliveira@icb.ufmg.br (D.M.T.O.); elborba@ufmg.br (E.L.B.)

**Keywords:** gametophytic self-incompatibility, mating systems, pollen tube ultrastructure, pre-zygotic barriers, programmed cell death

## Abstract

In most species of Pleurothallidinae, the self-incompatibility site occurs in the stylar canal inside the column, which is typical of gametophytic self-incompatibility (GSI). However, in some species of *Acianthera*, incompatible pollen tubes with anomalous morphology reach the ovary, as those are obstructed in the column. We investigated if a distinct self-incompatibility (SI) system is acting on the ovary of *A. johannensis*, which is a species with partial self-incompatibility, contrasting with a full SI species, *A. fabiobarrosii*. We analyzed the morphology and development of pollen tubes in the column, ovary, and fruit using light, epifluorescence, and transmission electron microscopy. Our results show that the main reaction site in *A. johannensis* is in the stylar canal inside the column, which was also recorded in *A. fabiobarrosii*. Morphological and cytological characteristics of the pollen tubes with obstructed growth in the column indicated a process of programmed cell death in these tubes, showing a possible GSI reaction. In addition, partially self-incompatible individuals of *A. johannensis* exhibit a second SI site in the ovary. We suggest that this self-incompatibility site in the ovary is only an extension of GSI that acts in the column, differing from the typical late-acting self-incompatibility system recorded in other plant groups.

## 1. Introduction

Homomorphic sporophytic self-incompatibility (SSI) and gametophytic self-incompatibility (GSI) systems are genetically controlled mechanisms capable of preventing fertilization by gametes from self-pollen [1,2]. Usually, they impede germination of pollen grains or the growth of incompatible pollen tubes in the stigma or style, respectively [1,2,3]. Another homomorphic self-incompatibility mechanism found in a few angiosperm families is the late-acting self-incompatibility system (LSI), where the self-incompatibility reaction acts in the ovary before the pollen tubes penetrate the ovules or after fertilization [4,5]. Although self-incompatibility (SI) has been recorded in species of various angiosperm families, relatively few studies have investigated the genetic control associated with SI in these species [5]. This is mainly due to difficulties in developing extensive diallel cross experiments between offspring, as is the case for Orchidaceae.

The presence of self-incompatibility in Orchidaceae, which is a family that is predominantly self-compatible, is mostly restricted to a few groups, such as species of *Dendrobium* C. Agardh [6], Oncidiinae [7], and Pleurothallidinae [8]. Since SI has not been genetically characterized in these groups, morphological aspects and the development of pollen grains and pollen tubes have been used to infer the type of SI in Pleurothallidinae [8,9,10,11,12]. Species of Pleurothallidinae (ca. 4.100 species) are predominantly fly pollinated [13,14,15] since most of the species studied showed some degree of self-incompatibility [8,9,10,11,12,16,17,18]. In this subtribe, different sites of occurrence of self-incompatibility reactions were mentioned in other species such as, in the stigma, where pollen grains do not germinate (similar to that observed in SSI, [10,11]), the stylar canal, where pollen tube growth is interrupted in the column/style (similar to GSI, [8,9]), and in the ovary, where the pollen tubes are obstructed before the ovules are penetrated [9,17].

Some species of *Acianthera* Scheidw. (Pleurothallidinae) are partially self-incompatible, resulting in a low fruit set incidence (10–13%) after self-pollination [8,9,12]. These fruits from self-pollination have high levels (ca. 80%) of seeds without the embryo, presenting only the seed coat or dead embryonic tissue [8,9,12]. Commonly, the high number of seeds without the embryo in fruits resulting from self-pollination has been explained as a possible consequence of inbreeding depression [8,9,19,20], which causes the death of zygotes and embryos [21,22]. However, in aborted and mature fruits from self-pollination in partially self-incompatible *Acianthera* species, the pollen tubes develop normally in the stylar canal inside the column but had obstructed growth when reaching the ovary [8,9]. Thus, Borba et al. [8,9] suggested that the self-incompatibility system could also act in the ovary of these species. In this case, fruit development and differentiation of the ovules continue due to the presence of hormones produced during the growth of the pollen tubes [23,24], which could explain the high levels of “empty structures”.

In this study, we investigated if a second self-incompatibility system occurs in the ovary of *Acianthera johannensis* (Barb.Rodr.) Pridgeon & M.W.Chase, which exhibits partial self-incompatibility. For this species, we analyzed the morphology and development of pollen grains after germination and during the growth of the pollen tubes in the stylar canal inside the column and ovary. As a comparison, we also analyzed *Acianthera fabiobarrosii* (Borba & Semir) F.Barros & F.Pinheiro, which has a self-incompatibility reaction that only acts in the column [9].

## 2. Results

### 2.1. Pollen Tube Development after Cross-Pollination

**Figure 1:**
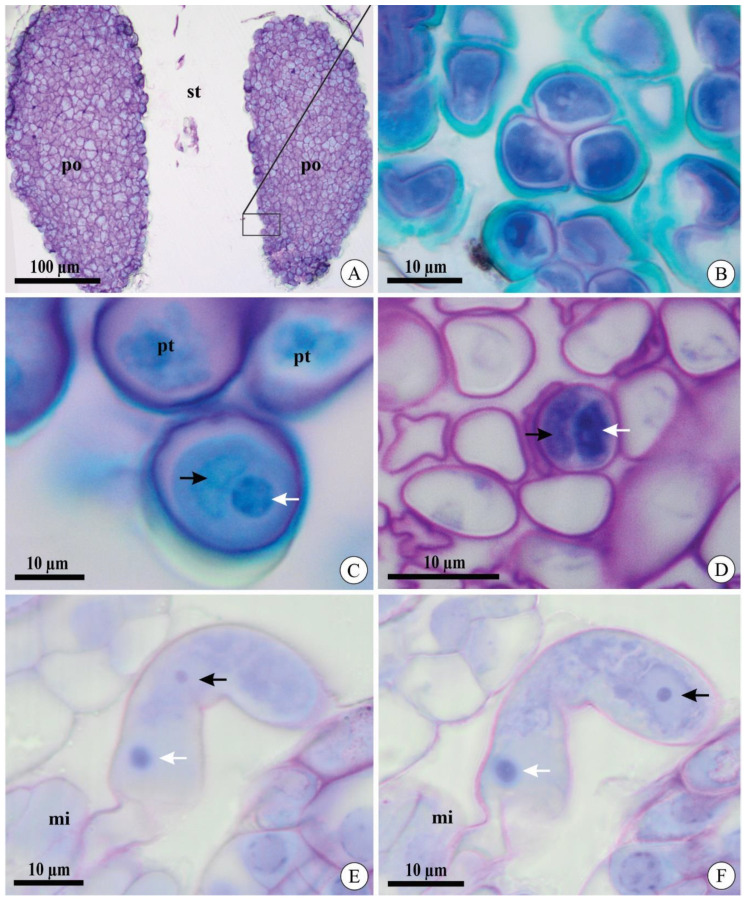
Pollinia and pollen tubes of *Acianthera johannensis* (**A**,**B**,**E**,**F**) and *A. fabiobarrosii* (**C**,**D**) under light microscopy, in transverse (**A**–**D**) and longitudinal (**E**,**F**) sections. (**A**) Two pollinia in the stigmatic cavity. (**B**) Pollen grain in tetrads. (**C**) Pollen grain after cross-pollination. Notice the generative cell (black arrow) and the nucleus of the vegetative cell (white arrow). (**D**) Pollen tubes after cross-pollination in the ovary 27 days after pollination (DAP). Notice the two cells in the microgametophyte, a generative cell (black arrow), and the nucleus of the vegetative cell (white arrow). (**E**,**F**) Pollen tube in the micropyle 40 DAP in two sequential focus planes. Notice the nucleus of the vegetative cell in both figures (white arrow) and two sperm cells (black arrows), with one visible in each figure. mi, micropyle. po, pollinia. pt, pollen tube. st, stigmatic cavity.

After cross-pollination, pollen grain germination and pollen tube morphology during growth were similar in *A. johannensis* and *A. fabiobarrosii*. Both species have two pollinia in their flowers, which are formed of pollen grains in tetrads (Figure 1A,B). At the germination time, the microgametophyte is bicellular and comprises a vegetative cell and a generative cell (Figure 1C). We also observed the bicellular microgametophyte during its growth in the stylar canal inside the column and ovary (Figure 1D). The two sperm cells were observed in a section in that the pollen tube penetrated the ovule (Figure 1E,F).

**Figure 2:**
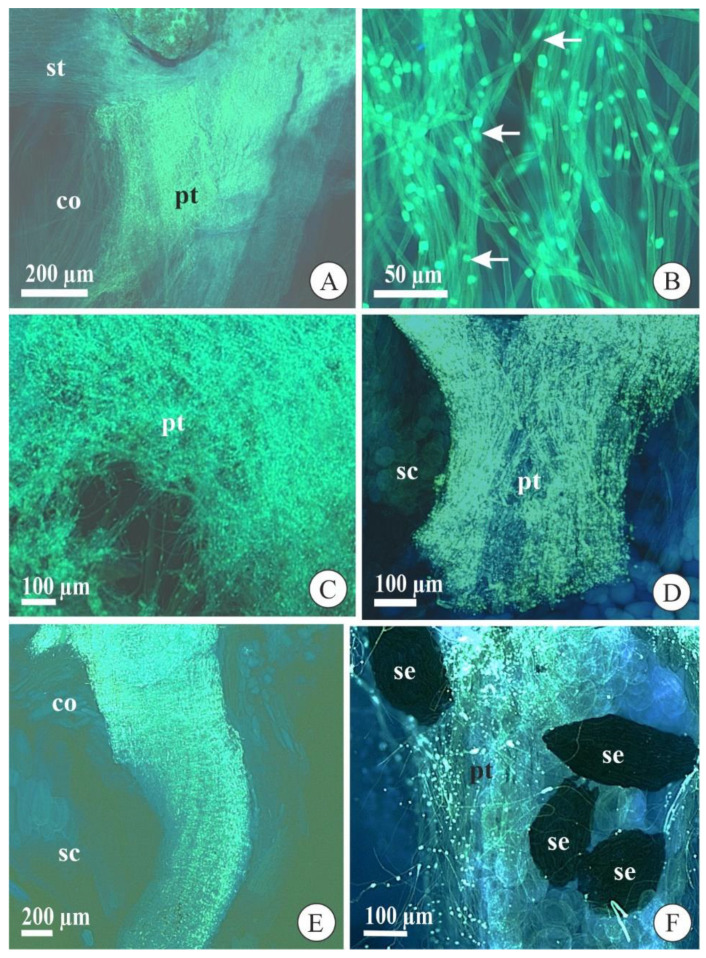
Pollen grain germination and pollen tube growth in flowers and fruits of *Acianthera fabiobarrosii* (**A**,**C**,**E**) and *A. johannensis* (**B**,**D**,**F**) observed with an epifluorescence microscope after cross-pollination. (**A**,**B**) Pollen tubes growing in the stylar canal inside the column (7 days after pollination-DAP). In B, observe the callose plugs regularly arranged along the length of the tube (arrows). (**C**) Pollen tubes reaching the ovary (10 DAP). (**D**) Pollen tubes in the seed chamber (38 DAP). (**E**) General view of pollen tubes in the column and seed chamber in a fruit (120 DAP). (**F**) Pollen tubes near the seeds with embryo in mature fruit (90 DAP). co, column. pt, pollen tube. sc, seed chamber. se, seed. st, stigmatic cavity.

In both species, the pollen grains deposited in the stigmatic cavity germinated with approximately three days after pollination (DAP) and started to grow along the stylar canal inside the column (Figure 2A). These pollen tubes had synchronized growth, that is, they grew together during their development until the ovary. Besides, the pollen tubes presented typical morphology with a uniform diameter and regular deposition of callose plugs along their lengths (Figure 2B). At approximately 10 DAP, the pollen tubes arrived at the column base and penetrated the ovary (Figure 2C). In the ovary, the pollen tubes separated into three bundles that went to each of the three placentae after 12 DAP, where it remained until ovule differentiation and maturation. These pollen tubes showed typical morphological aspects in the seed chamber of immature and mature fruits in both species (Figure 2D,F).

### 2.2. Pollen Tube Development after Self-Pollination

**Figure 3:**
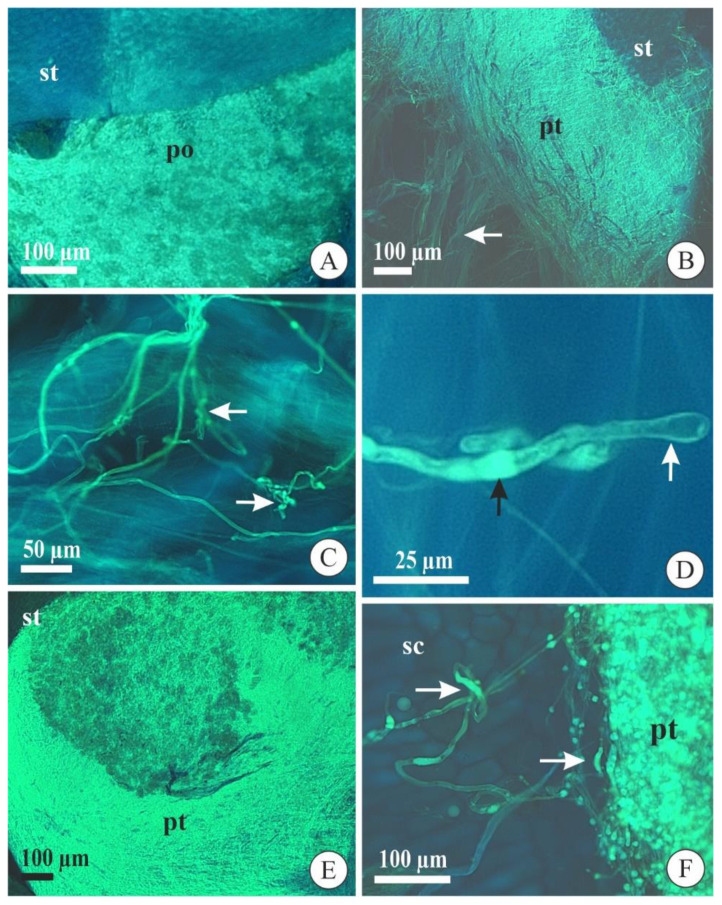
Pollen grain germination and pollen tube growth in flowers and fruits of *Acianthera fabiobarrosii* (**A**,**C**) and *A. johannensis* (**B**,**D**–**F**) observed with an epifluorescence microscope after self-pollination. (**A**) Pollinia in the stigmatic cavity (five days after pollination-DAP). (**B**) Pollen tubes (arrow) that stopped growing near the apex of the stylar canal inside the column (7 DAP). (**C**) Pollen tubes with a tortuous appearance (arrows) stopped growing in the column (7 DAP). (**D**) Detail of the incompatible pollen tube in the column. Observe the callose plug (black arrow) and the dilated tip (white arrow). (**E**) Pollen tubes in a withered self-pollinated flower from a partially incompatible individual (7 DAP). Note the synchronous growth of pollen tubes in the stylar canal. (**F**) Pollen tubes with regular morphology in mature fruit (90 DAP). Observe some pollen tubes with irregular patterns (arrows). po, pollinia. pt, pollen tube. sc, seed chamber. st, stigmatic cavity.

Pollen grain germination in *A. johannensis* and *A. fabiobarrosii* occurred three and five days after self-pollination, respectively, and it was still restricted to the stigmatic cavity at this time (Figure 3A). In aborted self-pollinated flowers (6–7 DAP), we observed pollen tubes near the middle of the stylar canal inside the column in both species (Figure 3B). Most of the aborted flowers of *A. johannensis* and all aborted flowers of *A. fabiobarrosii* from 7–14 days had pollen tubes with obstructed growth in the column base, and they did not reach the ovary. These pollen tubes did not grow synchronously throughout the stylar canal and were tortuous (Figure 3C) with irregular deposition of callose plugs and a dilated tip (Figure 3D), thus, presenting anomalous morphology. In some aborted flowers (10 DAP) of *A. johannensis*, the pollen tubes grew regularly and synchronously in the stylar canal inside the column (Figure 3E), which is similar to the growth of cross-pollinated pollen tubes. However, when they arrived at the locule, they became anomalous. We observed this process in the four individuals (of 28) that developed some fruits after self-pollination (*n* = 13 fruits of 101 self-pollinations in these individuals). Although compatible pollen tubes were obtained from cross-pollination, leading to fruit set with a highly viable seed rate, we also observed compatible pollen tubes in self-pollinated flowers, which developed fruits with bearing low of seeds with the embryo. These fruits contained regular and irregular pollen tubes in the seed chamber. Most pollen tubes in mature fruits of *A. johannensis* from self-pollination had normal morphology and regular deposition of callose plugs. However, some pollen tubes presented similar characteristics to those that stopped growing in the column (Figure 3F). Although the number of pollen tubes in the stylar canal inside the column and the ovary/fruit was not quantified, we could qualitatively observe a much higher number of pollen tubes after cross-pollination when compared to self-pollination.

### 2.3. Pollen Tube Ultrastructure after Experimental Pollination

**Figure 4:**
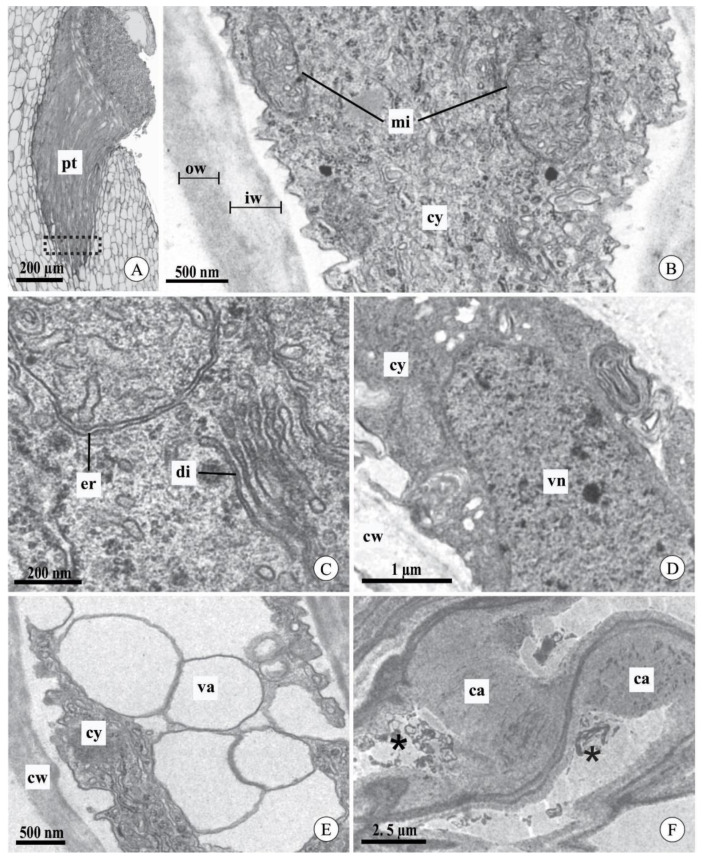
Pollen tubes in the stylar canal inside the column and seed chamber of fruits of *Acianthera johannensis* (**A**,**D**,**F**) and *A. fabiobarrosii* (**B**,**C**,**E**) after cross-pollination, using light microscopy (**A**) and transmission electron microscopy (**B**–**F**). (**A**) A general view of pollen tubes in the stylar canal inside the column. The traced rectangle is the region analyzed by transmission electron microscopy. (**B**) The apical region of the pollen tube with typical cytoplasm and cell wall with an inner layer of low electron density (light gray) and an outer layer of high electron density (dark gray). (**C**) Detail of the pollen tube with normal development showing abundant endoplasmic reticulum and dictyosomes. (**D**) Pollen tube showing the nucleus of the vegetative cell. (**E**) Distal region of a pollen tube with vacuoles of regular contour and hyaline content. (**F**) Pollen tubes in the seed chamber of mature fruit (90 days after pollination). Note the degenerated cytoplasm (asterisks) and the presence of callose. ca, callose. cw, cell wall. cy, cytoplasm. di, dictyosome. er, endoplasmic reticulum. iw, cell wall inner layer. mi, mitochondria. ow, cell wall outer layer. pt, pollen tube. va, vacuole. vn, vegetative cell nucleus.

We did not observe differences in the ultrastructural aspects of pollen tubes after cross-pollination between the studied species or between the intervals analyzed (Figure 4A–F). In general, the pollen tubes had a cell wall composed of two layers: an inner, light gray layer with low electron density, and an outer, dark layer with high electron density (Figure 4B). In the apical region of the pollen tubes, the cytoplasm had typical organelles, such as mitochondria with evident cristae (Figure 4B) and typical and well-developed dictyosomes and endoplasmic reticulum (Figure 4C). The nucleus of the vegetative cell was observed in the analyzed sections (Figure 4D). However, it was not possible to locate the sperm cells. The most distal region of the pollen tube had vacuoles with a regular contour and hyaline content (Figure 4E). In mature fruits, pollen tubes did not contain live cytoplasm and exhibited excessive callose deposition throughout most of their length (Figure 4F).

**Figure 5:**
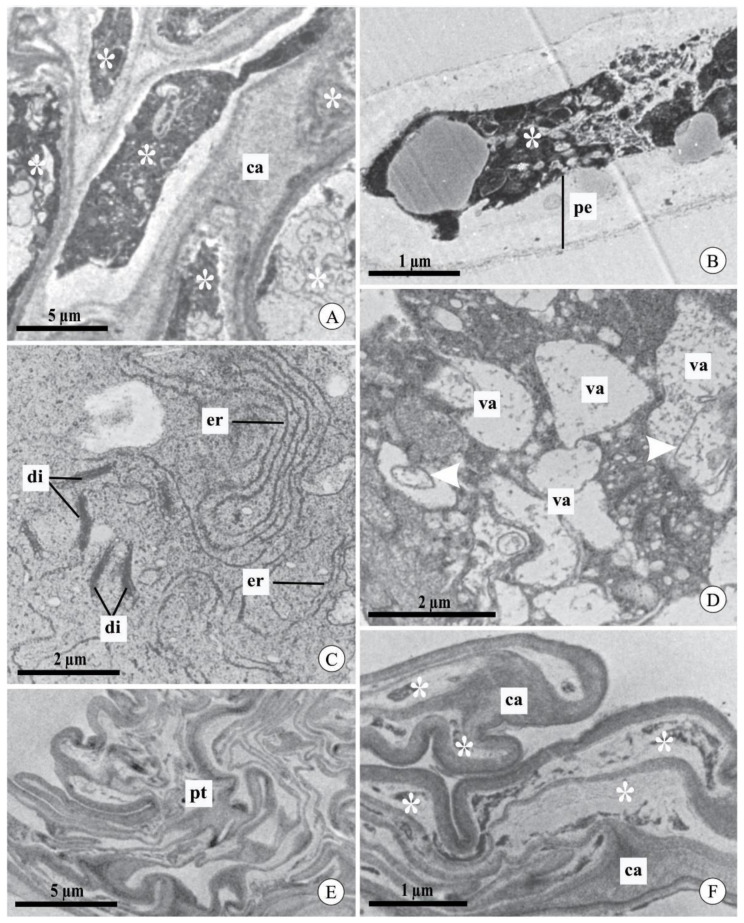
Pollen tubes in the stylar canal inside the column and seed chamber of fruits of *Acianthera johannensis* (**A**,**C**–**F**) and *A. fabiobarrosii* (**B**), after experimental self-pollination, using transmission electron microscopy. (**A**) Pollen tubes with the cytoplasm (asterisks) in a degeneration process (7 days after pollination-DAP), evidenced by the high electron density. (**B**) Pollen tube (9 DAP) with large periplasmic space and degenerated cytoplasm (asterisk). (**C**) Detail of a pollen tube with endoplasmic reticulum with dilated membranes and dictyosomes in the degeneration process (7 DAP). (**D**) Pollen tube with cytoplasm in the degeneration process. Note the presence of vacuoles with an irregular contour and flocculated content with internal membranes (arrowheads). (**E**,**F**) Pollen tubes in the seed chamber of mature fruit (90 DAP). In (**F**), notice the degenerated cytoplasm (asterisks) and the deposition of callose plugs. ca, callose. di, dictyosome. er, endoplasmic reticulum. pe, periplasmic space. pt, pollen tube. va, vacuole.

In contrast, in both species, self-pollinated flowers at 7 and 9 DAP mostly had degenerated pollen tubes in the stylar canal (Figure 5A,B). The first evidence of degeneration of the pollen tubes occurred in the organelles, as observed in the endoplasmic reticulum and dictyosomes, which had dilated and degenerated membranes, respectively (Figure 5C). Besides this, during the degeneration process, the vacuoles showed an irregular contour and flocculated content, including fragments of membranes (Figure 5D). Degeneration of the cytoplasm of the pollen tubes was demonstrated by its high electron density, with indistinct organelles and no visible nuclei (Figure 5B). The pollen tubes in mature fruits from self-pollination contained an excessive deposition of callose and degenerated cytoplasm (Figure 5E,F), which is similar to those observed in mature fruits from cross-pollination.

## 3. Discussion

The changes observed in incompatible pollen tubes of *Acianthera fabiobarrosii* and *A. johannensis*, at the morphological and cellular levels, corroborate the presence of a self-incompatibility reaction in the column, as pointed out by Borba et al. [8,9]. Although most SI reactions occur in the column of *A. johannensis* flowers, we could identify a second reaction site in the ovary of this species, which was observed in 22% of the individuals studied.

This second SI reaction site in the ovary differs from the typical late-acting self-incompatibility system in which the reaction occurs only in the ovary [4,5,25]. Therefore, the two self-incompatibility reaction sites exhibited in *A. johannensis* are likely governed by typical GSI, which is supported by the morphological uniformity observed in the incompatible pollen tubes in both sites. The GSI reaction extending to the ovary is rare but was reported for species in other families [4], as in *Camellia sinensis* L. (Theaceae), which, with the same genetic control, act in the style and ovary of different flowers [26]. In this sense, we interpreted the presence of anomalous pollen tubes at the ovary and fruit of partially self-incompatible individuals of *A. johannensis* as a delay of the GSI reaction.

Although we observed anomalous incompatible pollen tubes at the ovary of some individuals of *A. johannensis*, most of them were viable and fertilized 60% of the ovules after self-pollination [27]. Thus, the low number of seeds with embryos in fruits from self-pollination in this species is not directly related (or is not the only determinant) to the SI mechanism, but rather to inbreeding depression [27]. The self-incompatibility and inbreeding depression action in the same individual has been recorded mainly in species with pre-zygotic LSI [4,28,29] in which the ovule fertilization high rate after self-pollination is due to a failure in the SI system. Thus, the inbreeding depression is the cause of seed abortion after self-pollination in *A. johannensis* [27], which is a phenomenon that we can confirm in other partially self-incompatible Pleurothallidinae species, as reported by Borba et al. [8,9].

Pollen tube growth is a complex process that involves interactions with the pistil through chemical and molecular reactions [30,31,32,33]. However, when pollen tube growth is interrupted in the style by a self-incompatibility reaction, changes in the ultrastructure occur in the incompatible tubes [34,35,36]. These changes initially occur in organelles (e.g., dictyosome and endoplasmic reticulum), which is followed by the degeneration of the cytoplasm [34,35], as found in *A. johannensis* and *A. fabiobarrosii*. Cellular changes in incompatible pollen tubes were observed in species with GSI, such as *Papaver rhoeas* L. [35] and *Petunia hybrida* E. Vilm. [34,36], which are related to programmed cell death (PCD). However, the degradation process of vacuoles in incompatible pollen tubes was not recorded in these species with recognized GSI. This characteristic is usually observed in other plant cells, which also exhibit PCD [37]. Thus, we recognize that the change in vacuoles is related to PCD of incompatible pollen tubes in the species studied here, attributing more than one characteristic to this process. The typical GSI reactions that can lead to PCD in pollen tubes are based on S-RNase proteins in the pistil and F-box proteins in the microgametophyte in Solanaceae [5,38,39] as well as a Ca^2+^-dependent signaling cascade in Papaveraceae [40]. In the case of self-incompatible Orchidaceae species, molecular studies have not revealed the presence of S-RNases, suggesting that a distinct molecular control of GSI could occur in the family [41,42]. Additionally, there are no records involving a Ca^2+^-dependent signaling cascade in the death of pollen tubes in Orchidaceae.

In general, a different reaction mechanism of GSI could act in the death of incompatible pollen tubes in *A. johannensis* and *A. fabiobarrosii* as well as other orchid species. Although the molecular mechanism that acts in the death of pollen tubes after self-pollination in *Acianthera* is unknown, we suggest that this mechanism is involved in PCD in incompatible pollen tubes in the species we studied. Thus, molecular and diallel studies are necessary to elucidate the type of GSI reaction involved in the death of pollen tubes in the self-incompatible species of Orchidaceae.

## 4. Materials and Methods

*Acianthera johannensis* and *A. fabiobarrosii* are rupicolous species endemic to the *campos rupestres* in Minas Gerais State, Southeastern Brazil. Although these species exhibit high floral similarity and share the same pollinators, they do not occur in sympatry [43]. Both species flower from November to February, and anthesis lasts 7 to 10 days [43]. The process of megasporogenesis and megagametogenesis occurs only after pollination in these species [27,44]. Pollen tubes reach the ovary 7 DAP, the ovules are mature with approximately 20 DAP, and fertilized after 40 DAP [27,44]. *Acianthera johannensis* and *A. fabiobarrosii* fruits reach maturity three and four months after pollination, respectively [9,44].

We collected individuals of *A. johannensis* in the municipalities of Carrancas, Tiradentes, and São Tomé das Letras (*n* = 50), and individuals of *A. fabiobarrosii* in Joaquim Felício and Grão Mogol (*n* = 39). Posteriorly, we maintained these plants in greenhouses at the Departamento de Botânica at the Universidade Federal de Minas Gerais, Brazil. Vouchers are deposited in the herbarium at the Universidade Federal de Minas Gerais (BHCB; *A*. *fabiobarrosii*—Borba et al., 513, *A. johannensis*—Borba et al., 2230). We carried out controlled pollinations to confirm the presence of a self-incompatibility system in the greenhouse populations of each species.

We carried out experimental cross-pollination (*A. johannensis n* = 532, *A. fabiobarrosii n* = 134) and self-pollination (*A. johannensis n* = 418, *A. fabiobarrosii n* = 157) of flowers on the first day of anthesis to analyze the development of pollen tubes at different intervals of days after pollination. We collected flowers (1–14 DAP), immature fruits (15–60 DAP), and mature fruits (90–120 DAP) and fixed them for microscopy analyses. We considered compatible pollen tubes as those that exhibited normal development and morphology as well as incompatible pollen tubes as those that had anomalous growth and died in the stylar canal or ovary [8,9].

We fixed flowers (*n* = 26) and mature fruits (*n* = 18) in FAA (37% formaldehyde, acetic acid, 50% ethyl alcohol, 1:1:18 *v*/*v*, [45]) after experimental pollinations for light microscopy analyses. After embedding in synthetic resin (2-hydroxyethyl-methacrylate, Leica, Leica Microsystems, Heidelberg, Germany), we made longitudinal sections of the column and transverse sections of the ovary and fruit, at a thickness of 4.5–5 µm, with a Zeiss Hyrax M40 rotary microtome, and stained the sections with 0.05% toluidine blue in an acetate buffer at pH 4.7 (modified Reference [46]). To study these, we used an Olympus CX41 light microscope coupled to a camera.

The growth and morphology of pollen tubes after pollination were analyzed with fluorescence microscopy in flowers (self = 87, cross = 35), immature fruits (self = 3, cross = 9), and mature fruits (self = 4, cross = 10), fixed in FAA for 48 h and then preserved in 50% ethanol. We softened the samples in 10 N NaOH at 60 °C for 45 min (flowers) or 60 min (fruits), washed them in distilled water, and then stained them with 1% blue aniline (modified Reference [47]). The analyses were conducted with an epifluorescence microscope.

We selected samples of the column of self-pollinated and cross-pollinated flowers 7 and 10 DAP and mature fruits to analyze the pollen tube ultrastructure (*n* = 10). We fixed samples in Karnovsky fixative (pH 7.2 in 0.1 M phosphate buffer, modified [48]), post-fixed them in 1% osmium tetroxide (pH 7.2 in 0.1 M phosphate buffer), and processed them according to standard techniques [49]. We examined ultra-thin sections using a transmission electron microscope (Tecnai G2-Spirit, Philips/FEI Company, Eindhoven, The Netherlands) at 80 kV.
